# Evaluating the content and quality of intrapartum care in vaginal births: An example of a state hospital

**DOI:** 10.4274/tjod.88123

**Published:** 2017-03-15

**Authors:** Zekiye Karaçam, Döndü Arslan Kurnaz, Gizem Güneş

**Affiliations:** 1 Adnan Menderes University Faculty of Health Sciences, Division of Midwifery, Aydın, Turkey; 2 Aydın Maternity and Children’s Hospital, Clinic of Birth, Aydın, Turkey

**Keywords:** Intrapartum, care, quality of health care, Bologna score

## Abstract

**Objective:**

The purpose of the research was to assess the content and quality of the intrapartum care offered in vaginal births in Turkey, based on the example of a state hospital.

**Materials and Methods:**

This cross-sectional study was conducted between January 1^st^, 2013 and December 31^st^, 2014 at Aydın Maternity and Children’s Hospital. The study sample consisted of 303 women giving vaginal birth, who were recruited into the study using the method of convenience sampling. Research data were collected with a questionnaire created by the researchers and assessed using the Bologna score. Numbers and percentages were assessed in the data analysis.

**Results:**

The mean age of the women was 25.14±5.37 years and 40.5% had given one live birth. Of the women, 45.2% were admitted to hospital in the latent phase, 76.6% were administered an enema, 3.3% had epidural anesthesia, 2.6% delivered using vacuum extraction, and 54.1% underwent an episiotomy. Some 23.8% of the women experienced spontaneous laceration that needed sutures. The babies of two women exhibited an Apgar score below 7 in the fifth minute. When the quality of the intrapartum care given to the women was assessed with the Bologna score, it was found that 92.7% went into labor spontaneously, 100% of the births were supervised by midwives and doctors, 97.7% of the women had no supporting companion, and the nonsupine position was only used in 0.3% of the women. A partogram was used to follow up on the birth process in 72.6% of the women, and 82.5% achieved contact with their babies within the first hour after birth. Induction was applied in 76.6% of the women and fundal pressure in 27.4%.

**Conclusion:**

The study revealed that the quality of intrapartum care in vaginal births was inadequate. Reformulating the guidelines regarding intrapartum care in accordance with World Health Organization recommendations and evidence-based practices may contribute to improving mother and infant health.

## PRECIS:

Using the Bologna score, we evaluated the content and quality of the intrapartum care offered in vaginal births in Turkey, based on the example of a state hospital.

## INTRODUCTION

The high birth rate and percentages of maternal and infant deaths in Turkey continue to occupy a priority as major health issues. The World Health Organization (WHO)^(1)^ calls attention to the fact that the number of midwives, midwifery care outcomes, and quality are essential in reducing maternal and infant mortality rates and in reaching related global goals. Monk et al.^(2)^ reported that spontaneous vaginal birth rates are higher among women who give birth in maternity units that are under the direction of midwives and that newborn health indicators exhibit similar or even better outcomes when this is practiced. On the other hand, because statistics on maternal and infant mortality do not reflect the quality of maternal healthcare services, the process of caregiving must be evaluated separately^(3)^. In this context, the content and quality of intrapartum care is of vital importance in putting an end to mother and infant mortality and morbidity rates that are a consequence of preventable causes^(4)^.

Researches in recent years and evidence-based practices have led to important changes in intrapartum care. With the rise in the use of technological methods such as electronic fetal monitorization and induced labor, length of stay in the hospital has been reduced and the scope of midwifery, nursing, and medical care has changed^(5)^.

The strategies of the WHO^(6)^ designed to end preventable maternity mortality have pointed to the need to prioritize the basic care and follow-ups of women and newborns throughout the process of labor and birth. In addition, in the effort to end preventable maternal and infant mortality and morbidity, it is stressed that every pregnant woman and newborn needs to receive evidence-based basic care from well-trained healthcare professionals and to have this in a supportive environment^(4)^. From another perspective, research points to the probable association between intrapartum care practices and the rise in cesarean rates, so it is important to avoid elective induction practices and to maintain professional teamwork if cesarean rates are to be reduced^(7)^.

Intrapartum care constitutes an important part of healthcare directed toward women in Turkey. The technological developments of recent years have significantly affected the scope of intrapartum care. Elective cesarean and general cesarean section birth rates have steadily increased, rising to 48.1%^(8)^. Despite reports that epidural anesthesia raises the risk with cesareans^(9)^, there has been a rising trend toward the use of epidural anesthesia. At the same time, there has also been an increasing trend toward interventions with which restricted use is recommended, such as episiotomy (20%) and induction (10%)^(10,11)^. It is known that practices such as the lithotomy position (used in almost all births), unnecessary palpation, shaving of the perineum, enema, use of catheters, establishing vascular access, restriction of eating and drinking water, amniotomy (60%), and fetal monitorization are widespread^(11,12)^. Moreover, there are serious issues with providing emotional and physical support during labor and with practicing nonpharmaceutic pain management methods. These are matters about which adequate assistance is not provided. From a different perspective, however, there are some positive developments in Turkey in the context of intrapartum care. Almost all births (97.2%) take place in the hospital and with the assistance of healthcare professionals (97.4%)^(8)^. Rates of early bonding between mother and child and starting breastfeeding are considerably high (70.2%)^(13)^.

There are no studies in the national literature that address the content and quality of intrapartum care. There are certain criteria used in evaluating the quality of intrapartum care^(14)^. This study employed the standard measurement instrument, the Bologna score, which is based on the WHO recommendations for evaluating the quality of care in vaginal deliveries, which was drawn up by Chalmers and Porter^(15)^ and used by Sandin-Bojö and Kvist^(16,17,18)^. The purpose of this research was to assess the content and quality of intrapartum care offered in vaginal births in Turkey, based on the example of a state hospital and using the Bologna score.

## MATERIALS AND METHODS

The research was of cross-sectional design. The study was conducted between January 1^st^, 2013 and December 31^st^, 2014 at Aydın Maternity and Children’s Hospital with 303 women giving birth by vaginal delivery who were recruited on the basis of a convenience sampling. The number of vaginal births at this hospital in 2013 was 3051. The number of women recruited into the study was determined as 341 at a confidence level of 95% (s=0.05), with p=0.50 and n=3051. A total of 360 women who expected a vaginal delivery were invited into the study. Of these women, 48 were taken in for emergency cesarean section and data could not be collected for 9, resulting in the final analysis being performed with a total of 303 women. The study protocol was approved by the Adnan Menderes University Faculty of Medicine Ethics Committee (approval number: 2012/111).

In the 150-bed Aydın Maternity and Children’s Hospital where the research was conducted, midwives provide care for women going into vaginal delivery. At this hospital, women are generally placed in the lithotomy position, with their back slightly raised, their legs in stirrups and mediolateral episiotomy is performed, particularly on primigravidae. Moreover, the practice in this hospital proscribes vaginal delivery after cesarean section has been performed once. In the two-year period in which this research took in the hospital (2013-2014), the rate of primary cesarean births was 15.63% (n=1397/8937), the total cesarean rate was 31.08% (n=2778/8937), and the rate of operative delivery (vacuum) was 1.18% (n=73/6191).

The researchers collected data for the research using a questionnaire that was developed based on the pertinent literature^(19,20)^, and assessed using the Bologna score. The questionnaire’s 36 items probed the women’s sociodemographic and obstetric characteristics; whether their pregnancies had been planned and wanted; whether they had received antenatal care; their height and weight; smoking status; cervical dilation upon admittance to hospital; whether an enema, epidural anesthesia, vacuum or episiotomy had been performed during labor; and the baby’s Apgar score in the fifth minute.

Bologna score: This is an instrument that was developed by Chalmers and Porter^(15)^ for the purpose of assessing the quality of care given to women during the process of labor, based on the intrapartum care recommendations of the WHO^(21)^. The researchers first translated the Bologna score into the Turkish language. To verify the comprehensibility and the applicability of both the questionnaire and the Bologna score, a pilot study was conducted with 10 individuals, after which sections that were difficult to understand or apply were revised.

Women who were admitted to the maternity unit and were expected to have a vaginal delivery were invited into the study. The women were first informed about the study and their verbal and written consent was obtained. Later, the researchers completed the questionnaires based on the results obtained from face-to-face interviews held with the mothers. Other parts of the research data were obtained from patient files and through observations. All of the questions in the data collection instrument contained concrete data and therefore no differences stemming from observations existed.

### Statistical Analysis

The Statistical Package for the Social Sciences Version 18 (PASW Inc, Chicago, IL, USA) was used in the data analysis. All of the variables in the study were analyzed using descriptive statistics.

## RESULTS

The study was conducted with 303 women giving vaginal birth whose mean age was 25.14±5.37 years (range, 14-41 years). Data on the women’s age groups, their educational level and employment status, perceived income level, social security status, civil status, obstetric characteristics, body mass index (BMI) before pregnancy, and weight gained during pregnancy can be found in Table 1.

Of the women in the study, 35.7% (n=108) had experienced one, 29.1% (n=88) two, 20.1% (n=61) three, and 15.1% (n=61) had experienced 4-13 pregnancies; 40.5% (n=123) had gone through one live birth and had one living child. Some 17.5% (n=53) of the women had experienced one and 3.9% (n=12) 2-4 spontaneous abortions, and 3.3% (n=10) had experienced one and 1.3% (n=4) 2-3 curettages.

It was found that 24.4% (n=74) of the women had not planned the pregnancy and 8.3% (n=25) had not wanted the pregnancy, 1.3% (n=4) had not received prenatal care, and 7.2% (n=22) received prenatal care after the 13^th^ week of pregnancy. It was observed that the women’s pre-pregnancy mean BMI score was 23.14±3.41 kg/m^2^ (range, 15.99-34.67 kg/m^2^) and their average weight gain over the course of the pregnancy was 10±21 kg (range, 0-33 kg). Smoking prior to pregnancy was reported by 20.8% of the women and 13.2% said they smoked during pregnancy (Table 1).

Table 2 reveals data on the women’s deliveries and their status of being at high risk. Four percent (n=12) of the women had not reached term when they were admitted to the maternity unit, the babies were not in the vertex presentation in 3.3% (n=10), and 7.3% (n=22) did not undergo a spontaneous onset of labor. The fetal heart rate of two fetuses (0.7%) were not within normal boundaries (120-160 bpm). There was some kind of obstetric complication in previous births in 5.0% (n=15) of the women, and 11.2% (n=34) had complications in the present birth; 1.7% (n=5) had a medical condition that needed special care.

The following risk factors were observed in some women in the study: preterm labor (n=16), presentation anomaly (n=10), induced labor (n=22), fetal distress (n=2), meconium amniotic fluid (n=5), oligohydramnios (n=2), intrauterine exitus (n=1), placenta previa (n=1), gestational diabetes (n=1), and gestational hypertension (n=1). Furthermore, bleeding occurred in 4 women after birth and 1 woman developed a deep vaginal tear, and 2 women’s infants had Apgar scores below 7 in the fifth minute.

Some 45.2% (n=137) of the women were admitted to hospital with a cervical dilation of 1-3 cm, 76.6% (n=232) were administered enemas, 3.3% (n=10) had epidural anesthesia, vacuum extraction was performed on 2.6% (n=8), and an episiotomy was performed on 54.1% (n=164). Spontaneous lacerations that needed suturing were experienced by 23.8% (n=72) women. Twelve (4.0%) women gave birth to babies who had Apgar scores below 7 in the first minute, and two women’s babies (0.6%) had Apgar scores below 7 in the fifth minute (Table 2).

When the quality of the care given to the women was evaluated using the Bologna score, it was found that 92.7% (n=281) went into spontaneous labor and all of the births were assisted by midwives or doctors. Only 7 (2.3%) women had a supporting individual by her side and only 1 (0.3%) gave birth in a nonsupine position. A partogram was used to follow up on the birth process in 72.6% (n=220) women, and 82.5% (n=250) achieved contact with their babies within the first hour after birth. Induction was used in 76.6% (n=232) of women and fundal pressure was applied to 27.4% (n=83) (Table 3).

## DISCUSSION

This study examined the content and quality of intrapartum care provided at a state hospital in Turkey using the Bologna score. From the observations of current practices of intrapartum care at the hospital, it could be seen that the use of a partogram, fundal pressure, and the nonsupine position, as well as having a supporting person present at the birth were not in compliance with the international standards recommended by the WHO nor with evidence-based practices. The data obtained are important in that they reveal the current status through the example of a hospital in Turkey.

It was seen that high-risk factors were at play in the case of some of the women in the study. This may have increased the probability that certain interventions such as induction would be performed.

A small percentage of the women (4.0%) were not at term (37+0-41+6 weeks) when they were admitted to the maternity unit for vaginal labor. A study conducted in Switzerland similarly noted that only 6.0% of pregnant women were admitted for delivery before their 37^th^ gestational week^(18)^. These outcomes may be associated with early delivery risk or with early membrane rupture.

A significant percentage woman (45.2%) was admitted to hospital in the latent phase. It is reported, however, that women should be admitted to the maternity unit at a later stage, that is, in the active phase of labor (when cervical dilation is 4 cm or more)^(22)^. It has also been asserted that early admittance to the maternity unit may be associated with increased rates of oxytocin and analgesic use^(22)^.

Our results could be associated with the traditional approach adopted by healthcare professionals and the general public in Turkey. Women are unable to distinguish between real and false labor, are unaware of the course of labor, and rush to the hospital as soon as they feel the first contractions because of the current inadequacy of prenatal education in Turkey. When this happens, healthcare professionals admit them to the hospital against the probability of risk, which occurred with most women (76.6%) in the present study. This rate is considerably higher than what was reported in Switzerland^(17)^ and Canada^(23)^, where rates were 39% and 5.4%, respectively. This might be a consequence of the country’s prevailing general health practices related to this matter. This is the case even though it is known in Turkey, however, that evidence-based research has shown that enemas and other applications that are not mother-friendly should be avoided under all circumstances.

It was observed in this study that epidural anesthesia was administered to only a few women (3.3%). This rate is much lower than reported by Sandin-Bojö et al.^(17,18)^, Chalmers et al.^(24)^, and Li et al.^(25)^ (57%, 48.7%, 57.3%, and 18.3%, respectively). This finding may be connected to the fact that techniques of coping with labor pains are not widely used in Turkey.

The vacuum technique is generally used as a birthing instrument in Turkey. The use of vacuum extraction in this study was 2.6%, lower than reported by Li et al.^(25)^ and Sandin-Bojö and Kvist^(18)^ (10.5% and 8.1%, respectively). In a study conducted in Canada, Chalmers et al.^(23)^ reported a vacuum extraction utilization rate of 10.0% and a rate of 14.3% for all instrumental deliveries. In another study performed at the same location as the present study, the vacuum extraction rate was similar (3.74%), significantly lower than reported in studies conducted in other countries^(11)^. Although the low rate of births with intervention reported in the literature in Turkey is a positive finding, this may be associated with the high rate of cesarean deliveries.

It was observed in the study that episiotomies are still widely implemented (54.1%). Similar results were reported in studies previously conducted in Aydın (59.21%) and Ankara (64.0%)^(11,12)^. These rates are much higher than reported in the United Kingdom (UK)^(25)^ and Canada^(24)^ (11.2% and 20.7%, respectively). This finding is important because it reflects the traditional approach of clinical personnel to performing episiotomies.

A significant percentage of women (23.8%) experienced spontaneous lacerations that needed suturing. In another study that was carried out previously at the same hospital, spontaneous lacerations requiring repair with sutures were reported as 8.98%^(11)^. This rate was reported as 64% in a study in Canada^(24)^ and 15.7% in the UK^(25)^. The differences in these results may be associated with birth assistance techniques used by health professionals (e.g., birth position, perineal massage, management of the second phase).

When intrapartum care was evaluated in this study using the Bologna score, it was found that most women (92.7%) experienced spontaneous onset of labor. The study encompassed all births in the hospital and all of these births were attended to by midwives or doctors. On the other hand, the Turkish Ministry of Health encourages hospital births in the presence of health professionals. The Turkish Population Health Research Survey results of the last five years show that 97% of the births taking place in the country occurred under the supervision of health professionals^(8)^. Similarly, studies conducted in Brazil^(26)^ and Switzerland^(18)^ reported that all births occurred with the help of health professionals. These findings are positive and gratifying when considered in terms of maternal and infant health.

Studies on the provision support during childbirth in Turkey are still controversial and all intrapartum care is handled by midwives. The results of the present study confirmed that only 7 women were able to receive supportive intrapartum care. The WHO^(1)^, on the other hand, strongly recommends in its “Coalition for Improving Maternity Services”^(10)^ that among the mother-friendly care that can be provided, mothers in childbirth should have, besides midwives, nurses, and doctors on hand, a doula (a woman assisting the mother in labor) or other individuals (spouse, partner, family member, friend) to provide support during labor. In their study in Switzerland, Sandin-Bojö and Kvist^(18)^ reported that 98.7% of mothers in labor received this kind of support. This brings the matter of intrapartum care services in Turkey to the forefront and points to the need for reviewing and developing the system.

Similar to other Turkish studies^(11,12)^, the present study observed that almost all births occurred with women in the supine position and her legs in stirrups. This high percentage is pronounced when compared with the rate of 35% reported in Switzerland^(18)^ and the rate of 57% reported in Canada for placing women’s legs in stirrups and the rate of 48% for the use of the supine position^(24)^. In other studies, it is reported that even though placing the mother in vertical positions during labor and childbirth positively affects the mother’s control and satisfaction in terms of the length of the second stage, the performance of an episiotomy, instrumental birth, severe pain, and the fetal heartbeat^(27)^, it is the attitudes of health professions that play a decisive role in choosing a position for the mother^(28)^. Nieuwenhuijze et al.^(29)^ stressed that making a joint decision is important in terms of increasing women’s sense of control and satisfaction.

In this study, most births (73%) were monitored using a partogram. Rani et al.^(30)^ and Sandin-Bojö and Kvist^(18)^ reported similar findings (72% and 93%, respectively). Giglio et al.^(26)^, however, reported a considerably lower percentage (29%). According to these results, it may be said that the use of the partogram varies depending upon the individual working protocols of institutions.

In most births (83%), contact between mother and child took place in the first hour after birth. Similar results were reported in other studies conducted in Turkey^(13,31)^. This rate was 92% in a study conducted in Switzerland^(18)^. These findings show that healthcare practices are satisfactory in terms of ensuring early contact between mother and child.

In this study, the rate of inducing labor was significantly high (76.6%). Similar results have been reported in other studies conducted in Turkey^(11)^. In studies carried out in many other countries, lower rates for using oxytocin or labor augmentation were reported^(17,18,23,24,25,26)^. Moreover, the WHO^(1)^ suggests as part of its description of mother-friendly hospital applications that the practice of induced labor must be used to a limited extent and not for more than 10% of births. These findings indicate that there is a need to carry out studies that will help to reduce the use of induction methods in Turkey.

The WHO^(32)^ reports a very low quality of evidence regarding the routine use of amniotomy and induction and offers a weak recommendation regarding these procedures. Smyth et al.^(33)^ also asserted that amniotomy was not recommended as a standard part of intrapartum care and management. In the present study, however, and similar to the results of other studies conducted in Turkey^(11)^, it was observed that amniotomy (67%) and induction (60%) were widely used. Matsuo et al.^(34)^ and Sartore et al.^(35)^ reported lower rates of induction in their studies (49% and 22%, respectively). These findings are important in that they show that amniotomies and induction are in fact routine in Turkey.

It was seen in the study that fundal pressure was applied to a significant percentage (27.4%) of women. This rate is lower than previously reported in other studies conducted in Turkey^(11,36)^. On the other hand, it is higher than reported in other countries^(18,23)^. This finding is significant because it points to the dimension of the wide use of fundal pressure in intrapartum care in Turkey despite the recommendation that this is not made a routine part of care given in this period.

### Study Limitations

There are some limitations to this study. The first is that the study was based on a nonrandom sampling. Accordingly, the results obtained represent the women taken into the study and cannot be generalized. Secondly, the study data were collected through face-to-face interviews and observations from patient files. The different sources may have affected the reliability of the data obtained. Only concrete and uncomplicated data may increase the reliability of research. Thirdly, at the hospital where the study was carried out, all cases of spontaneous lacerations were sutured. This may have explained the higher percentage of sutured lacerations compared with that reported in the international literature. Fourthly, the difficulty in collecting data in the study for cases of emergency and planned cesareans made it necessary to include only women who were having their babies through vaginal delivery. Accordingly, there is a need for studies that include emergency and planned cesarean births. This study does, however, include the ratios of primary and total cesarean deliveries that took place over the period the research was conducted.

## CONCLUSION

This study revealed that the intrapartum care provided at a state hospital in Turkey was in compliance with international standards and evidence-based practices recommended by the WHO in terms of the presence of trained health professionals during labor and that early bonding was achieved between mother and child. However, the care was not in compliance in terms of factors related with admittance to hospital, performing episiotomies, enema, the use of partograms, induction, fundal pressure, and the nonsupine position, or in terms of receiving the assistance of a supportive individual during labor. According to the findings, recommendations might be: (1) Administrators should reformulate intrapartum care services to comply with the recommendations of the WHO, international standards and evidence-based practices. (2) The curriculums of programs of formal and widespread education should be reviewed and adjusted in concordance with standards and health professionals should thus be encouraged to update their knowledge and practices in this context. (3) Health administrators should create institutional and national policies that will raise the quality of current healthcare services and bring them up to par with international standards. (4) Studies on this matter should be conducted at different hospitals, with the inclusion of emergency and planned cesarean cases and with different sample groups. (5) More experimental, qualitative and quantitative research should be undertaken to explore specific problems at different hospitals to reveal the attitudes and experiences of healthcare professionals, and to suggest solutions.

## Figures and Tables

**Table 1:**
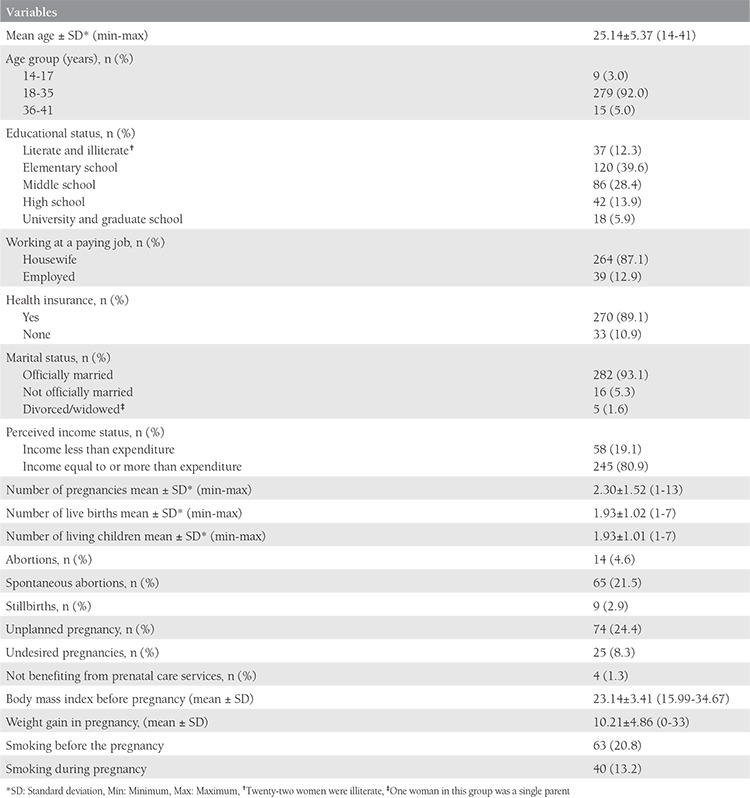
Sociodemographic characteristics of the women and variables related to pregnancy (n=303)

**Table 2:**
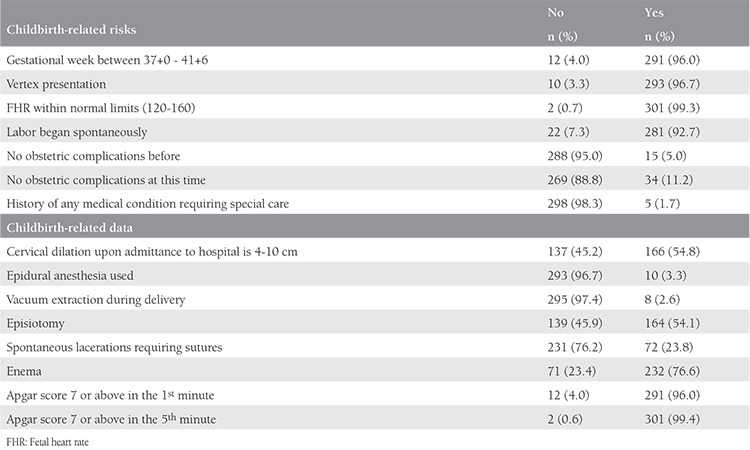
Risk status related to childbirth (n=303)

**Table 3:**
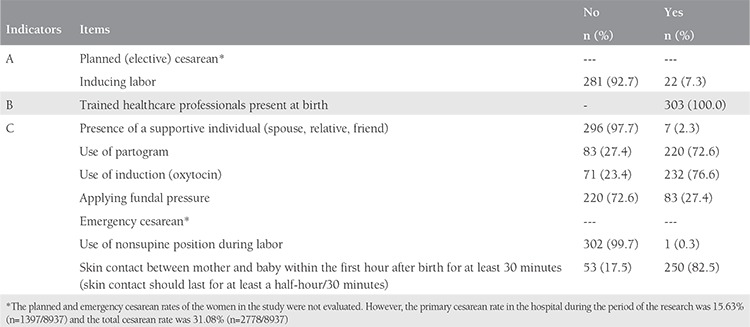
The items in the Bologna score (indicators of the effectiveness of care during labor) (n=303)
